# Supplementary material for: Development of a Scale to Measure Healthy Behaviors in Spanish-Speaking University Students
Source: Int J Environ Res Public Health. 2023 Feb 1;20(3):2627. doi: 10.3390/ijerph20032627 (PMC9915417; doi:10.3390/ijerph20032627)
Supplement: Supplementary file 1 [file ijerph-20-02627-s001.zip › ijerph-2090796-supplementary.pdf]

Table S1. Attitude to Healthy Behavior Questionnaire

Following are a series of ideas and/or beliefs, indicate your degree of agreement or disagreement with them, where:				
**Statement**	**Strongly disagree**	**Moderately disagree**	**Moderately agree**	**Strongly agree**
Evito comer en exceso (I avoid overeating)				
Mantengo una dieta balanceada (I maintain a balanced diet)				
Suelo consumir alimentos bajos en azúcar (I tend to eat foods low in sugar)				
Procuro comer frutas y verduras a diario (I try to eat fruits and vegetables daily)				
Evito consumir bebidas azucaradas (I avoid sugary drinks)				
**Statement**	**Strongly disagree**	**Moderately disagree**	**Moderately agree**	**Strongly agree**
Realizo ejercicio constantemente (I exercise constantly)				
Me ejercito todas las semanas (I exercise every week)				
Procuro mantener rutinas de ejercicio (I try to maintain exercise routines)				
Hago actividad física dos o más veces por semana (I do physical activity two or more times a week)				

Strongly disagree = 1 and Strongly agree =4
